# Immobilization of Glycoside Hydrolase Families GH1, GH13, and GH70: State of the Art and Perspectives

**DOI:** 10.3390/molecules21081074

**Published:** 2016-08-17

**Authors:** Natália G. Graebin, Jéssie da N. Schöffer, Diandra de Andrades, Plinho F. Hertz, Marco A. Z. Ayub, Rafael C. Rodrigues

**Affiliations:** Biotechnology, Bioprocess, and Biocatalysis Group, Food Science and Technology Institute, Federal University of Rio Grande do Sul, Av. Bento Gonçalves 9500, P.O. Box 15090, ZC 91501-970 Porto Alegre, RS, Brazil; natigraebin@gmail.com (N.G.G.); jessieschoffer@gmail.com (J.d.N.S.); dcandrades@gmail.com (D.d.A.); plinho@ufrgs.br (P.F.H.); mazayub@ufrgs.br (M.A.Z.A.)

**Keywords:** enzyme immobilization, glycoside hydrolases, β-glucosidase, α-amylase, cyclodextrin glycosyltransferase, dextransucrase

## Abstract

Glycoside hydrolases (GH) are enzymes capable to hydrolyze the glycosidic bond between two carbohydrates or even between a carbohydrate and a non-carbohydrate moiety. Because of the increasing interest for industrial applications of these enzymes, the immobilization of GH has become an important development in order to improve its activity, stability, as well as the possibility of its reuse in batch reactions and in continuous processes. In this review, we focus on the broad aspects of immobilization of enzymes from the specific GH families. A brief introduction on methods of enzyme immobilization is presented, discussing some advantages and drawbacks of this technology. We then review the state of the art of enzyme immobilization of families GH1, GH13, and GH70, with special attention on the enzymes β-glucosidase, α-amylase, cyclodextrin glycosyltransferase, and dextransucrase. In each case, the immobilization protocols are evaluated considering their positive and negative aspects. Finally, the perspectives on new immobilization methods are briefly presented.

## 1. Introduction

Carbohydrates are important natural molecules presented in their free moieties or in association in glycoproteins, glycolipids, and polysaccharides, playing fundamental roles in the cell physiology and development of all organisms [1]. The enzymes that cleave or, inversely, mediate the ligation of glycosidic bonds of glycoconjugates, oligosaccharides, and polysaccharides can be classified by two different systems. The IUBMB Enzyme Nomenclature of the Enzyme Commission (EC) is based on distinct enzymatic activities, substrate specificity and occasionally on their molecular mechanism [2]. In this system, the EC 3.2.1 comprises the enzymes that hydrolyze O- and S-glycosyl compounds and comprises enzymes from EC 3.2.1.1 trough EC 3.2.1.196, with some deletions and reclassifications. The second system of enzyme classifications is based on the carbohydrate-active enzymes (CAZy) database (http://www.cazy.org/), where the enzymes are classified into several families based on their amino acid sequence similarities [3,4]. Currently, the Glycoside Hydrolases (GH) family classification from CAZy extends from GH1 to GH135, with 190 different enzyme activities (based on the EC code), mainly glycosidases from EC 3.2.1, but also some glycotransferases from EC 2.4.1.

Among the enzymes from the GH families, some, such as amylase, cellulase, pectinase, hemicellulase, glucansucrase, lactase, invertase, and β-glucosidase, are of great interest to various industries. These enzymes are well studied in the literature and there are several reviews dealing with their catalytic mechanism and properties [5,6,7,8,9].

Because of their biological origin and function in live organisms, enzymes are sometimes unsuitable for direct industrial process applications. In natural systems, enzymes are usually soluble, are inhibited by substrates and products, show low stability, and do not possess an ideal catalytic characteristic when applied to non-natural substrates. These disadvantages can be overcome by their immobilization in appropriate supports and matrices [10,11]. Enzyme immobilization is defined as the process of confinement of the enzyme molecule into or onto a phase (matrix/support) different from that of substrates and products [12]. Immobilized enzymes can be applied in different reactor configurations, allowing easy reaction control, avoiding product contamination of the enzyme, which is an important property in food technology allowing their reuse over many reaction cycles [10]. Additionally, immobilization can improve biocatalyst stability and to modify the enzymatic activity, specificity, as well as enantio and regioselectivities [13].

Considering all these aspects, the objective of this review is to discuss on the protocols used for immobilization of enzymes from the GH families GH1, GH13, and GH70, more specifically β-glucosidases, α-amylase, cyclodextrin glycosyltransferase, and dextransucrases. Initially, we present an overview on the types of immobilization techniques, and then proceed to cover the state of the art of enzyme immobilization of the aforementioned family of enzymes. Finally, we discuss on the perspectives of new developments in the field of enzyme immobilization for these three GH families.

## 2. Types of Enzyme Immobilization

Enzyme immobilization methods can be divided into three categories: enzyme molecule attachment to a solid support, entrapment into a matrix, and molecule cross-linking [11].

**Figure 1:**
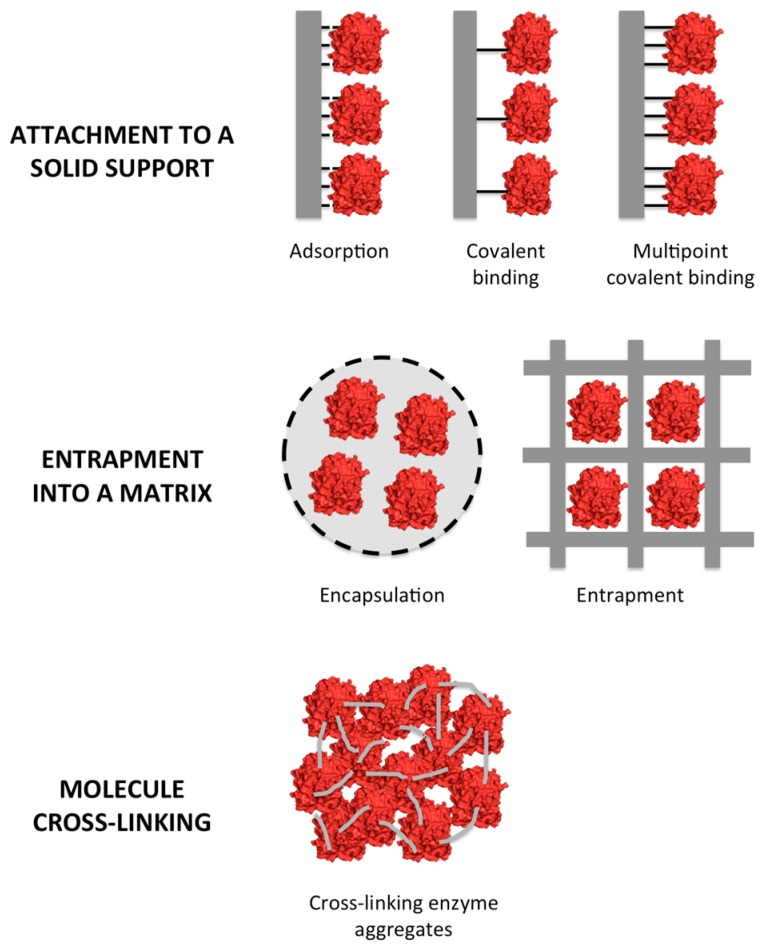
Types of immobilization.

The attachment to a solid support can be reversible or irreversible, and can be achieved using a broad variety of chemical and physical methods. Entrapment consists in the retention of enzyme molecules into a polymer matrix by covalent or non-covalent bonds. Cross-linking of enzymes employs bi-functional reagents to prepare carrier-free macroparticles [12,14,15,16,17,18,19,20,21,22]. A schematic representation of the types of immobilization is presented in Figure 1.

There are several reviews dealing with enzyme immobilization, explaining in details all advantages and disadvantages of each protocol [10,11,12,17,18,19,22,23,24], chemical [25,26,27], and genetic [28,29] modifications of enzymes in order to improve immobilization, one-step purification and immobilization of enzymes [30], stabilization of protein quaternary structure by immobilization [31], the effect of different supports on enzyme properties [32,33,34,35,36], as well as the different enzymatic reactors using immobilized enzymes [37,38].

Considering that the focus of this review is to discuss on the immobilization of glycoside hydrolases and the fact that methods of immobilization have been extensively reviewed, in this paper we will attain to present the basic concepts of each type of immobilization, as mentioned before, in order to better elucidate the discussion that will follow.

### 2.1. Enzyme Attachment to a Solid Support

The use of pre-existing supports for enzyme immobilization is possibly the most used technique. As mentioned before, the enzyme attachment to a solid support can be reversible or irreversible. The cost of the support will impact the overall cost of the final biocatalyst, thus must be considered during the design of the biocatalyst [10]. The reversible immobilization is achieved by adsorption of the protein on the surface of the support [14]. Adsorption uses physical interactions between the support and enzyme, including van der Waals forces, hydrophobic and ionic interactions, and hydrogen bonding. The binding is generally weak and does not change the native structure of the enzyme.

Irreversible immobilization is obtained by covalent linkage of the enzyme to the support. The reaction occurs between amino acids on the enzyme surface and reactive groups placed on the support surface, being glutaraldehyde the reactive chemical most used for this covalent immobilization of the enzyme [39]. Glutaraldehyde is a bi-functional reagent that is used to activate the support and also to react with the enzyme, generally involving primary amine groups of the protein, although it may eventually also react with other groups (thiols, phenols, and imidazoles). Two other groups are frequently used to stabilize the 3D structure of the enzyme by multipoint covalent attachment: glyoxyl and epoxide [40,41]. Both groups can react with primary amine groups of Lys at pH 10, promoting several linkages between enzyme and support, which produces high stabilization factors when compared to the soluble enzyme [13,20].

The use of adsorption presents the advantage of support recovery after the loss of enzyme activity, allowing the immobilization of new enzyme load. However, there is the risk of enzyme leakage and product contamination by the enzyme. On the other hand, covalent attachment usually provides better stability than adsorption, specially using multipoint covalent attachment. Nevertheless, when the enzyme losses its activity, all preparation (enzyme plus support) should be discarded.

### 2.2. Entrapment

The technique of entrapment consists in the inclusion of an enzyme molecule into a polymer network, such as an organic polymer or a silica sol-gel, or a membrane device, such as a hollow-fiber or a microcapsule. The physical restraints are generally weak to prevent the enzyme leakage. Therefore, additional covalent attachment is often required. Entrapment generally requires the synthesis of the polymeric matrix in the presence of the enzyme [11,22]. Entrapment also protects enzymes from the direct contact with the environment, minimizing the effects of gas bubbles, mechanical shear, and hydrophobic solvents, but it has the drawback of mass transfer limitations and producing low enzyme loadings [17].

A common method of entrapment consists in the use of silica sol–gel matrices formed by hydrolytic polymerization. The polymer porosity, its network structure, surface functionalities, and particle size, can all be modified by adjusting the polymerization conditions [42].

Alginate and chitosan are other two natural polymers that are often used for enzyme entrapment. Chitosan can be applied to obtain covalent immobilizations, however, the enzyme may also be entrapped into the gel if mixed with chitosan prior to the cross-linking reaction [43]. Finally, entrapment by nanostructured supports like electrospun nanofibers and pristine materials are being tested, making possible the application of immobilized enzymes in a wide-range of biocatalytic processes in the field of fine chemistry, biomedicine, biosensors, and biofuels [12].

The main advantage of the entrapment is the physical protection against harsh conditions, such as high temperature, extreme pH, gas bubbles, and the use of solvents in the reaction medium. In the opposite, the barrier formed between the enzyme and the carrier may promote diffusional limitations, leading to low productivities in the system.

### 2.3. Cross-Linking of Enzymes

Cross-linked enzyme crystals (CLECs) or aggregates (CLEAs) are prepared by using a bi-functional reagent to obtain carrier-free macroparticles. This approach offers some advantages: highly concentrated enzyme activity in the catalyst is achieved, showing high stability and with low production costs owing to the exclusion of an additional carrier [11,22]. CLECs are based on the crystallization of pure enzymes, followed by their chemical cross-linking, whereas CLEAs preparation involves the enzyme aggregation before chemical cross-linking [19,22,44]. CLEAs are generally preferred over CLECs because CLEAs do not require the purification of enzymes and allow the ease co-immobilization of several enzymes [25]. CLEAs are prepared by the precipitation of the enzyme, or the mixture of enzymes, using organic solvents (e.g., acetone, ethanol, propanol, *tert*-butanol), salts (e.g., ammonium sulfate), or non-ionic polymers (e.g., polyethylene glycol) [11]. Next, the aggregates are cross-linked using bi-functional reagents, usually glutaraldehyde as it is inexpensive and commercially available [39], although other cross-linkers, such as dextran aldehyde, have been successfully used in cases where glutaraldehyde presented poor results [45].

In some instances, the addition of a proteic feeder, such as bovine serum albumin, can be used to improve the cross-linking when the target protein is poor in Lys residues [46,47,48]. Additionally, magnetic CLEAs (mCLEAs) can be prepared by performing the cross-linking in the presence of functionalized magnetic nanoparticles. These mCLEAs can be separated by magnetic precipitation or can be used in a magnetically stabilized fluidized-bed reactor [49,50,51,52].

CLEAs present the advantage of absence of solid support, where all solid particle is protein, increasing the productivity in terms of mass of protein by solid area, and consequently, decreasing the costs of immobilization process. However, the enzyme is more exposed to reaction medium compared to the other protocols, being more sensible to possible denaturation caused by high temperature, pH, gas bubbles or some other inactivation agent.

## 3. The GH1 Family of Enzymes

The GH1 glycoside hydrolase enzyme family includes, in the CAZy database, more than 9900 enzymes from the eubacteria kingdom (approximately 90%). There are also enzymes from archaea, fungi, plants, and animals. Belonging to the GH1 family, 23 different EC numbers are found, represented by β-mannosidase (EC 3.2.1.25), β-glucuronidase (EC 3.2.1.31), β-xylosidase (EC 3.2.1.37), β-d-fucosidase (EC 3.2.1.38), 6-phospho-β-glucosidase (EC 3.2.1.86), 6-phospho-β-galactosidase (EC 3.2.1.85), and lactase (EC 3.2.1.108). However, the β-glucosidases (EC 3.2.1.21) and β-galactosidases (EC 3.2.1.23) represent the main enzymes in this family according to their importance [53].

**Figure 2:**
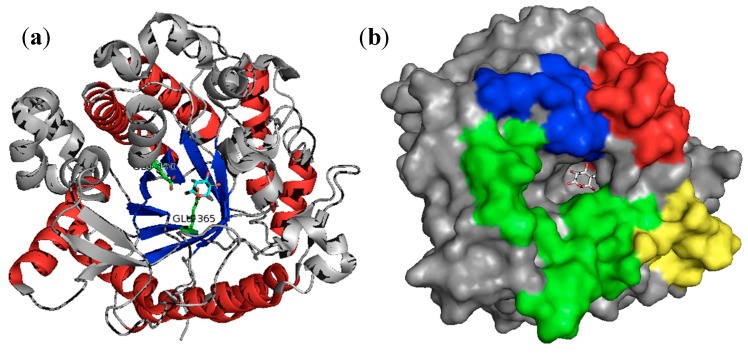
Structure of GH1 β-glucosidase complexed with gluconolactone. (**a**) Ribbon representation of the (α/β)_8_-TIM barrel structure and active site. α-Helices are shown in red and β-sheet in blue. Glu170 and Glu165 are represented by green lines; (**b**) Molecular surface with representation of four loops on the entrance of active site. Loops A, B, C and D are shown in red, blue, green and yellow, respectively. The 3D structure was obtained from the Protein Data Bank (PDB) using Pymol vs. 0.99. The PDB code is 2E40.

In general, the GH1 enzymes have a classical (α/β)_8_-TIM barrel fold structure that contains their active site. The hydrolysis of the glycosidic bond is catalyzed following the β-retaining action mechanism by two amino acid residues of the enzyme: a glutamate residue as catalytic proton donor and another glutamate residue as catalytic nucleophile/base [54,55]. The catalytic residues are highly conserved among other families that constitute GH clans, such as families GH13 and GH70. In particular, glutamate acts as a nucleophile in enzymes from GH1 family, characterizing them in the GH-A clan [56,57]. The family presents the nucleophile located close to the carboxy-terminus from β-strand 7 and a sequence of asparagine-glutamate (an asparagine residue preceding the general acid/base catalyst) close to the carboxy-terminus from β-strand 4, except for myrosinase, where the acid/base glutamate is replaced by glutamine [58,59]. Henrissat et al. [60] suggested that the two key active site glutamic acids are about 200 amino acid residues apart from each other and these enzymes are able to hydrolyze a wide diversity of substrates with a similar disposition of their identical catalytic residues. The structure of β-glucosidases from GH1 family is represented in Figure 2 by the isoform A from *Phanerochaete chrysosporium* complexed with gluconolactone (PDB: 2E40) [61]. The catalytic residues are Glu365 (nucleophile) and Glu170 (acid–base), located in the center of the (α/β)_8_-TIM barrel structure (Figure 2a). Additionally, the entrance of the substrate-binding pocket is formed primarily by four extended loops connecting strands and helices at the C terminal side of the barrel (Figure 2b).

Although the high diversity of enzymes in the GH1 family, in this review, we will discuss aspects of the immobilization of β-glucosidases only, since these constitute some of the most important enzymes from the industrial point of view.

### 3.1. β-Glucosidases Features

The β-glucosidases (β-d-glucoside glucohydrolase, EC 3.2.1.21) are found in Archaea, Eubacteria, and Eukaryotes, playing several functions in these organisms, as glycolipid and exogenous glycoside metabolism in animals, mechanism defense, cell wall lignification, release of aromatic compounds in plants, and biomass conversion in microorganisms [62,63]. These enzymes catalyze the hydrolysis of β-glycosidic bonds between a monosaccharide and a moiety, which may be a carbohydrate or not [63,64]. The hydrolysis reaction can be divided into two steps. The first one involves the nucleophile attack in the anomeric carbon (C-1) of the substrate resulting in a covalent glycosyl–enzyme intermediate with concomitant release of the aglycone after the protonation of the glucosidic oxygen by the acid catalyst, step called glycosylation. The second step corresponds to the hydrolysis of the covalent intermediate glycosyl–enzyme, with the acid catalyst acting as a base and a water molecule functioning as the nucleophile, releasing the glucose and regenerating the nucleophile residue [58,65].

Regarding the β-glucosidases active site, it is divided into several subsites. The subsite, denominated subsite −1 or glycone subsite, is responsible for binding the non-reducing end of the monosaccharide of the substrate, whereas the remaining part of the substrate interacts with the aglycone-binding site that may be formed by several subsites (+1, +2, +3, +n), also called aglycone subsite. Three-dimensional structures of β-glucosidases with different substrates and inhibitors showed that the substrate cleavage point is between the subsites −1 and +1 [64,66,67].

β-Glucosidases are extensively studied enzymes for the applications in food, feed, textile, and paper industries [68]. For example, β-glucosidases have been used for ethanol production in the process of saccharification [69], in the improvement of aromatic flavor compounds in juices and beverages [70], and for the hydrolysis of non-starch polysaccharides used in the feed of monogastric animals [71]. For these industrial applications, it is required that the enzymes possess some characteristics, such as resistance to environmental conditions, namely, pH and temperature, and multiple reusability. They also have to be economic viable. The immobilization process can promote these desired characteristics [43].

### 3.2. β-Glucosidases Immobilization

Several techniques have been developed for β-glucosidases immobilization, including adsorption, entrapment in gels and membranes, covalent linkage to insoluble supports, and cross-linking with bi-functional reagents [72]. One of the first reports about immobilized β-glucosidases was published in 1974. The enzyme was covalently immobilized on cyanogen bromide-activated cellulose, and its catalytic properties decreased after immobilization [73]. In the following sections, we will present and discuss the several methods used to immobilize β-glucosidases, discussing about their advantages and drawbacks.

#### 3.2.1. Immobilization on Chitosan Particles

The covalent immobilization of β-glucosidases on chitosan is the most common method of immobilization for these enzymes (Table 1). This process was used to immobilize β-glucosidase in order to improve the aromatic potential of wines by cross-linking the enzyme using glutaraldehyde [74]. Immobilized β-glucosidases on chitosan beads for industrial application showed higher activities in a wider range of pH and temperatures, enhancing thermal stability, storage stability, and reusability, when compared to the free β-glucosidase [75]). Desai et al. [76] studied the immobilization of β-glucosidase from *Scytalidium lignicola* on chitosan. The authors prepared a biocatalyst showing higher resistance to temperature and pH values, without activity loss, and the immobilized enzyme retained 50% of its residual activity after five use cycles. In another research, Bissett and Sternberg [77], immobilized the β-glucosidase from *Aspergillus phoenicis* QM 329 on chitosan using glutaraldehyde as cross-linker. The immobilized enzyme exhibited similar pH optimum, but was more active at lower pH values, and improved thermal stability compared to free enzyme.

The cross-linked chitosan beads have innumerous advantages, such as an excellent hydrophilicity, high porosity, and large adhesion area. However, there are some operational limitations. Chitosan density is similar to water, causing it to easily float easily, and its texture is very soft, which limits its industrial applications. Aiming to eliminate these undesirable characteristics, it was performed the addition of activated clay to the wet chitosan (without freeze-drying) or dried chitosan (freeze-dried), followed by cross-linking [78]. In a similar way, activated carbon was added to chitosan beads before the cross-linking with glutaraldehyde [79]. The immobilization of β-glucosidase from *Exiguobacterium* sp. exhibited higher hydrolyzing activity of isoflavone glycoside, as well as higher pH and thermal stabilities in aqueous–organic two-phase system after these modifications of the support [79]. The same operational characteristics were obtained when chitosan was used as a base for magnetic carriers, when testing its potential recycling use in the hydrolysis of lignocellulosic biomass [80].

Another aspect concerning this support is the fact that the cross-linked chitosan microspheres exhibit reactive primary amino groups directly bonded with pyranoid rings, which causes large steric hindrances. The cross-linking reaction can consume a large portion of amino groups, reducing the enzyme load and the volumetric productivity. To improve these aspects, it was performed a cross-linking of chitosan modified by l-lysine (named LMCCR), where the Lys acted as flexible spacer arm to decrease the steric hindrances. The ε-NH_2_ of Lys residues replaced the less reactive amino groups of cross-linked chitosan during the cross-linking reaction.

The immobilized β-glucosidase showed optimal pH in alkaline region, had 85% of its residual activity after 13 cycles of use and efficiently produced resveratrol by the hydrolysis of polydatin in a continuous reactor [94,110].

#### 3.2.2. Covalent Immobilization of β-Glucosidases on other Supports

The covalent immobilization of β-glucosidases by direct linkage or cross-linking was also performed using other supports than chitosan. In addition, the cross-linking using glutaraldehyde has also been extensively employed in solid supports. Ahmed et al. [72] immobilized β-glucosidase from *A. niger* on sponge, which is safe, inexpensive, and readily available, by covalent binding it with glutaraldehyde as spacer group, obtaining high immobilization yields (95.67%), high activity recovery (63.66%), and a thermal stability 132-fold higher at 65 °C, compared to the free enzyme. The immobilization of β-glucosidase using spent coffee grounds as the solid carrier, cross-linked with glutaraldehyde, was also studied for the conversion of isoflavone glycosides into their aglycones in black soymilk. The results showed that the immobilized enzyme could be used for more than 30 cycles, with the enzyme retaining its catalytic activity for 20 days. According to the authors, these advantages enabled a less costly process [90].

Another support that has been widely studied as carrier in the immobilization of β-glucosidase via covalent binding is silica gel. Jung et al. [87], studied the immobilization of β-glucosidase using silica gel. In order to prevent the formation of covalent bonds near to the active site, the authors bonded β-glucosidase with cellobiose and glucose, which resulted in 176% higher enzyme activity, compared with the non-pretreated. In addition, the silica gel-immobilized enzyme kept 80% of its relative activity after 20 reuses. Agrawal et al. [84] improved the overall storage, pH, and temperature stability, and the length of reuse to up to 10 cycles with 70% of residual activity by immobilization of β-glucosidase from *Bacillus subtilis* on SiO_2_ nanoparticles. Singh et al. [111] also reported an increase of 288-fold in the thermal stability at 65 °C of this enzyme immobilized on SiO_2_ nanoparticles. Similar results were also obtained when β-glucosidase from *A. niger* was immobilized on magnetic nanoparticles functionalized with glutaraldehyde [91].

The covalent immobilization of β-glucosidase onto epoxy activated Eupergit C has also been reported. This macroporous carrier has the ability of stabilizing protein conformation forming very stable covalent multipoint attachments with amino, hydroxyl, thiol and/or phenolic groups of amino acid side chains on the enzyme surface. The bonds between the enzyme and support are highly chemically and mechanically stable during storage over a pH range from 2 to 12 [20]. The immobilization process using these particles improved the pH and storage stabilities of β-glucosidase from *Issatchenkia terricola* [98]; it also improved stability at 65 °C and the apparent K_m_ and V_max_ of β-glucosidase from *A. niger* [100]. One of the disadvantages of the covalent attachment is that the linkages can promote steric hindrances of the enzyme. In this way, a spacer arm can be added by modification of the support by ethylenediamine and glutaraldehyde to produce an aldehyde-activated support [112]. Khan et al. [99], showed that the reaction of epoxy group followed by reaction with glutaraldehyde in β-glucosidase from *Thermotoga neapolitana*, improved the thermal and the storage stabilities of the enzyme, and allowed extensive reuse of the biocatalyst, which kept 91% of the initial enzyme activity after 10 batch cycles. The same improved enzymatic characteristics were observed when β-glucosidase was immobilized using amine-epoxy agarose support [89]. 

Spagna et al. [86] reported studies of the immobilization of β-glucosidase onto an amine agarose gel. This immobilization occurred via oxidation of the carbohydrate chains of the enzyme, since they are not directly involved in the catalysis, thus the aldehyde groups were capable of reacting with the amine groups of the matrix and with their subsequent reduction. Before the reduction reaction, the formation of covalent bonds by activation of carboxyl groups with carbodiimide was also tested in order to increase the density of the covalent bonds for each enzyme molecule immobilized, thus increasing the rigidity of the secondary and tertiary structure of the enzyme. Although the immobilized enzyme exhibited high immobilization yields and enhanced stability, the activation of the biocatalyst using carbodiimide slightly negatively affected its activity.

In the light of these considerations, it is possible to propose that covalent binding provides the strongest attachment of the enzyme to the support, and it has been associated with high activities and the possibility of enzyme reuses. However, covalent attachment is generally achieved using complex chemicals as linkers, in multi-step reactions that require relatively long times to be completed [113]. Aiming to circumvent these disadvantages, Hirsh et al. [108] obtained a highly effective method for rapid covalent immobilization (within one minute reaction) using plasma immersion ion implantation (PIII) for activation of polystyrene film polymer surface. According to the authors, the immobilized thermophilic *Caldicellulosiruptor saccharolyticus* β-glucosidase showed an activity more than 20-fold higher than the commercial β-glucosidase, over five batch reuses. In another report, Nosworthy et al. [109], demonstrated that granules and particles of polystyrene treated with PIII were also successfully employed for immobilization, and the protein was strongly immobilized on the surface without the need of chemical treatments. This covalent binding enabled more robust linkages allowing high flow rates, high activity, large surface area and a broad operating pH range, making it possible its use into current reactor technologies, including batch, fluidized bed, and continuous flow reactors [108,109].

#### 3.2.3. Immobilization of β-Glucosidases by Adsorption

Although the immobilization by covalent bond could result in preparations showing high stability, the immobilization by physical adsorption can enhance the flexibility of the system. Table 2 presents the main reactive groups and supports used for β-glucosidase immobilization by adsorption.

Non-covalent immobilization methods have the advantages of being simple and low-cost, and do not employ severe conditions. These advantages of adsorption were demonstrated in the immobilization of β-glucosidase on polyacrylic resin activated by carboxyl groups applied to the hydrolysis of sugarcane bagasse [105]. Similar results were obtained in the synthesis of *n-*octyl-β-d-glucopyranoside by β-glucosidase immobilized on Amberlite XAD-4 [126] and in water-soluble polymer Eudragit S-100 [120]. Furthermore, adsorbed-immobilized β-glucosidase was applied in packed and fluidized bed reactors to improve the aromatic quality of Muscat wine and to the hydrolysis of cellulose [117,119]. In both cases, the enzyme was immobilized on ion exchange resins Duolite A-568 [117] and Amberlite DP-I [119].

Another similar application was the preparation of a mixed bed ion exchanger hydroxyapatite (HTP) formed by calcium phosphate, which was very useful for the purification and immobilization in a one-step procedure of several proteins, including β-glucosidase. The HTP-bound β-glucosidase was active, stable, and easily recoverable from reaction medium. This preparation was used for the enhanced release of aromas in wine and fruit juices [118].

The β-glucosidase adsorption on DEAE-sepharose showed to be high, around 91% of immobilization yields and 83% of activity recovered [121]. Using similar supports, β-glucosidase adsorption on MANAE-agarose and DEAE-cellulose were approximately 75 and 120-fold more stable than the free enzyme [123], and were quickly immobilized showing a high residual activity after immobilization. Moreover, it was possible to modify the protein, by different reactions, such as acylation of the amino groups by pyromellitic dianhydride (PMDA), increasing the negative charges on the protein surface, minimizing the time of the immobilization. Tyagi and Gupta [122], were able to immobilize the β-glucosidase in DEAE-cellulose resin after modification with PMDA. This chemical modification improved the thermal stability, and this strategy may be useful for obtaining enzyme derivatives for reversible adsorption on anion exchangers.

Magnetic nanoparticles are other carriers that were studied for the non-covalent immobilization of enzymes. Chen et al. [125] synthesized magnetic Fe_3_O_4_ nanoparticles coupled with agarose using co-precipitation via alkaline conditions and span-80 surfactants in organic solvent. The enzyme bounded efficiently via metal ion affinity in alkaline amino groups of its surface and the Co^2+^ chelated on the carriers, showing higher hydrolytic activity and higher thermal and operational stabilities than the free form. These nanoparticles can be easily separated from the reaction medium by magnetic field and it is possible to reuse them. In another study, β-glucosidase was immobilized onto Fe_3_O_4_ nanoparticles coated with sodium citrate and after was cross-linked with glutaraldehyde. This method presented extended ranges of pH and temperature activities, higher accessibility to the substrate (K_m_ value of immobilized β-glucosidase was lower than that of the free enzyme), high activity recovery (89%), and improved thermal and storage stabilities [75]. However, one disadvantage of using chemical supports, as cited early, is their high cost and possible environmental concerns during their discharges. The use of natural carriers could minimize these problems. In this way, towel gourd vegetable sponges were tested to immobilize a marine *A. niger* β-glucosidase. This carrier is natural, biodegradable, has a low-cost and it is safe for humans [116]. Other interesting natural materials are fine soil colloidal particles with high surface area and content of iron oxides were employed to immobilize β-glucosidase via adsorption. The immobilized enzyme showed thermal stability at all tested temperatures and it was less sensitive to pH and temperature changes than the free enzyme, possibly because the support presented a protective effect [115].

#### 3.2.4. Immobilization of β-Glucosidases by Entrapment

The entrapment in calcium alginate is the most frequently method used for β-glucosidase immobilization reported in the literature [70,95,131,132,133,134,135] (Table 3). This method showed good enzymatic recovered activities of 60% [134], 66% [136], and 73% [137]. However, when the whole cells from *Debaryomyces hanseniiwere* showing β-glucosidase activity were immobilized, the recovered activity was only 8% [133]. Although this immobilization method generally provides low mechanical strengths, the immobilization process produces enhanced thermal properties and higher optimum temperature usage [133,134,137], the possibility of reuse [134,137], and higher storage stability [133,134], compared to the free enzyme. The leakage of the enzyme out of the alginate beads causing its loss increases the immobilization costs, which is a disadvantage. In order to overcome this problem, some studies have been conducted to cross-link the enzyme with glutaraldehyde prior to the immobilization [135,138]. For instance, Su et al. [70] studied the calcium alginate as the carrier and cross-linking–entrapment–cross-linking as the immobilizing method to hydrolyze the glycosidic aroma precursors in tea beverages. The immobilized β-glucosidase exhibited optimum temperature 10 °C lower, a recovered activity of 46%, increased thermal and pH stabilities, and lower K_m_ than the free enzyme.

In addition to calcium alginate, enzyme entrapment has also been reported using other carriers, such as the hydrogels of poly(2-hydroxyethyl methactylate) that showed excellent protective effect [143]. Recently, Javed et al. [144] studied the β-glucosidase immobilization within nanoscale polymeric materials (polyurethane, latex, and silicone) and obtained good results using latex (highest relative activity) and silicone matrix (highest entrapment efficiency). However, the latex immobilized enzyme leaked after each cycle, which did not occur when the entrapment was in silicone. Figueira et al. [142] obtained good results using polyvinyl alcohol (PVA-) based matrices (Lentikats) and in sol-gel (prepared with tetramethoxysilane), analyzing retention of the catalytic activity following immobilization. The immobilization in sol-gel resulted in higher stability under higher operational temperatures compared to the immobilization using only Lentikats, because these particles were not physically stable above 55 °C.

The sol-gel matrices have the advantage of preventing leakage of enzyme from support during the reaction. However, the disadvantage of the gel contraction during condensation and drying process, causing possible enzyme denaturation, is known. To overcome this limitation, some mechanisms have been developed. For instance, Vila-Real et al. [119], studied the addition of ionic liquids in the sol–gel immobilization process. The authors improved the immobilization efficiency of the encapsulated enzyme, as well as the mechanical resistance against cracking, suggesting that the ionic liquids play an important role in enzyme performance [145].

One particularity of the β-glucosidases is that its industrial applications are usually associated with other enzymes to achieve a broader objective. In winemaking, for example, other enzymes can be co-immobilized in order to improve desirable effects, such as aroma and crystallinity. Co-immobilizations were studied and a simple and cost-effective procedure for the co-immobilization of β-d-glucosidase, α-l-arabinofuranosidase, α-l-rhamnopyranosidase, and β-d-xylopyranosidase was proposed by Ferner et al. [103]. The four enzymes were immobilized onto magnetic beads and showed good stability under winemaking conditions, with β-glucosidase showing the highest immobilization yields of 95% between pH 3.5 and 4.0. According to the authors, the immobilization method was easy to obtain, it was effective and the commercial preparation did not require cleanup steps [103].

Because β-glucosidase immobilization by adsorption or by entrapment causes a limited half-life of the biocatalyst due to progressive release of the enzyme into the reaction milieu, its applications are hampered. On the other hand, the covalent immobilization requires several chemical steps, which are frequently associated to substantial loss of enzyme activity [93]. In order to overcome these problems, Mateo et al. [20] studied the physical aggregation of enzymes followed by cross-linking (cross-linked enzyme aggregates, CLEAs) as a method to prepare solid biocatalysts. Using this method, Reshmi and Sugunan [93] obtained β-glucosidase immobilized onto mesocellular silica foams (MCFs) by formation of CLEAs of nanometer scale. The CLEAs retained activity over wider ranges of temperature and pH applications, and lower K_m_ than the free enzyme, and they were recyclable up to 10 cycles with more than 85% residual activity, with high enzyme loadings.

In conclusion, it is possible to observe that β-glucosidases have been intensively investigated regarding possible carriers and immobilization methods. The choice of immobilization technique should be dictated by the enzyme application, since it has been demonstrated that different immobilization protocols lead to unique characteristics. The search for novel matrices and immobilization strategies can help to overcome these obstacles.

## 4. The GH13 Family of Enzymes

**Figure 3:**
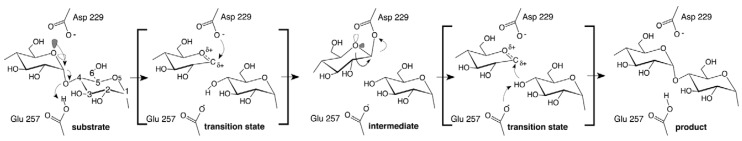
Mechanism of the α-retaining α(1-4) glycosidic bond cleavage used by the *α*-amylase family. Adapted from [149].

The GH13 enzyme family is the largest sequence-based family of glycoside hydrolases. It comprises a group of enzymes with different specificities, in which each one acts upon one type of substrate, composed by glucose residues linked through α(1-1), α(1-4), or α(1-6) glycosidic bonds. This family can be divided into two subgroups: the starch-hydrolyzing enzymes, and the starch-modifying or transglycosylation enzymes [146]. Hydrolases and transferases from GH13 family are multidomain proteins, sharing a common catalytic domain in the form of a (α/β)_8_ barrel fold: 8 parallel β-strands and 8 α-helices, being alternated along the protein sequence. The β-strands form the inner barrel, whereas the α-helices flank the exterior [147,148]. Enzymatic hydrolysis of the glycosidic bonds takes place via general acid catalysis that requires two critical residues: a proton donor and a nucleophile/base [54]. These enzymes have a retaining mechanism (Figure 3), acting in two steps: the glycosylation step, involving the formation of a covalent glycosyl-enzyme intermediate, and the deglycosylation, when hydrolysis occurs [54,146,147].

The seven most conserved amino acid residues in the *α*-amylase family cluster are precisely in the center of the regions that comprises the catalytic site. Three of them are totally conserved, Asp229, Glu257, and Asp328, whereas Asp135, His140, Arg227, and His327 are almost completely conserved. The evolutionary conservation of these residues can be explained by their common substrate, starch, which contains only one basic chemical bond, the *α*-glycosidic bond [149].

Transglycosidases share structural and sequence similarities to retaining glycosidases, as well as mechanistic strategies. However, instead of catalyzing the hydrolysis of glycosidic linkages, they utilize a sugar rather than a water molecule to act in the final step, yielding a new glycoside linkage (transglycosylation reaction) [150]. Cyclodextrin glycosyltransferases, members of GH13 family, use *α*-linked glucose polymers as substrates for the formation of cyclic oligoglucosides.

### 4.1. α-Amylases

Amylases are among the most studied and important enzymes used in industry [148]. These starch-converting enzymes are applied in the production of maltodextrin, modified starches, or glucose and fructose syrups. α-Amylases (α-1,4-glucan-4-glucanohydrolase; E.C.3.2.1.1), which are classified into the GH13 family, are extra-cellular enzymes that specifically catalyze the hydrolysis of α-1,4-glycosidic linkages of starch yielding low molecular weight products, such as glucose, maltose and maltotriose units; however, they do not hydrolyze β-1,6-glycosidic bonds [151,152].

These enzymes are found throughout natural sources, including plants, animals, and microorganisms, but their commercial production has generally been carried out using submerged fermentation using bacteria from the genus *Bacillus,* such as *B. licheniformis*, *B. stearothermophilus*, and *B. amyloliquefaciens* [152,153]. A large number of microbial amylases are commercially available and they have almost completely replaced chemical hydrolysis in the starch processing industries [154]. Furthermore, α-amylases have potential application in a wide number of industrial processes related to the food, fermentation, textile, paper, detergent, and pharmaceutical industries [146,155,156]. The properties of each α-amylase regarding thermostability, pH profile, pH stability, and Ca-dependency are important in the development of these processes.

#### 4.1.1. Immobilization of α-Amylases

α-Amylases from different sources have been immobilized onto a wide variety of organic and inorganic supports, among them, gelatin [157], gums [158,159], magnetic nanoparticles [160,161], silica [162], and carrier-free systems [163], increasing the enzymatic stability and adaptability to hard conditions of reactions.

- Immobilization of α-amylases on insoluble supports:

Covalent immobilization is the most used technique to attach α-amylases on solid supports. The covalent bond formed between the protein and the support usually shifts the optimal temperature of the enzyme, decreases its conformational flexibility and protects its structure from distortion or damage by heat exchange. Rana et al. [164] immobilized *α*-amylase onto chitosan microspheres using glutaraldehyde as chemical agent. The optimum catalytic temperature shifted from 45 to 55 °C presenting 70% more activity than its free form, at this temperature. The immobilized α-amylase retained 49% of its initial activity after seven consecutive batch reuses. When compared with Amberlite MB-150 (a mixture of acidic cationic and basic anionic resin) [165], the optimum pH obtained was 8.0 for chitosan beads and 7.0 for this inorganic support, while the free enzyme showed an optimum pH of 5.5. Changes in optimum temperatures were not observed for α-amylase immobilized onto chitosan derivative, contrasting with a small increase for Amberlite. The immobilized enzyme showed a good operational stability by retaining 38% and 58% of its initial activity after 10 cycles for chitosan and Amberlite, respectively. When attached to DEAE-cellulose, α-amylase retained 77% of its initial activity after 10 uses and had a change in the optimal pH, being 5.0 for soluble and 6.0 for immobilized enzyme. Immobilized and soluble enzymes showed optima activities at 70 °C and 68 °C, respectively [166]. On the other hand, Shukla [167] activated DEAE-cellulose with glutaraldehyde and found that α-amylase immobilized by covalent attachment to this support improved its optimal temperature, from 60 °C to 70 °C. The maxima activities of free and immobilized enzymes were observed at pH 7.0. The improved activity, at higher temperatures, in between 40 °C and 60 °C, was also observed by Veesar et al. [168], studying the immobilized α-amylase onto calix[4]arene activated with glutaraldehyde [169].

- CLEAs of α-amylases:

The costs of immobilized enzymes should be minimized in order to increase their competitiveness for technical applications, and CLEAs have emerged as a versatile carrier-free immobilization technique. Several researchers have already reported studies on this type of immobilization applied to α-amylases. These aggregates are formed by adding a precipitant agent, such as salt or organic solvent, followed by chemical cross-linking using a bifunctional reagent, usually glutaraldehyde [11,19,39,170,171]. It was also evaluated the effect of using non-toxic and biocompatible polysaccharides such as agar, chitosan, dextran, and gum arabic as cross-linkers on *α*-amylase recovered activity replacing the traditional glutaraldehyde on CLEAs production [172]. The macromolecular CLEA of α-amylase using dextran showed 91% of activity recovery, 84% using chitosan, whereas using glutaraldehyde, it was possible to recover only 42% of activity. The authors indicated that glutaraldehyde CLEAs had lower recovered activity owing to the compact supramolecular structure formation, resulting in serious steric hindrance effects. This may restrict the diffusion of macromolecular substrate inside the CLEA particle decreasing the catalytic activity [172]. According to Talekar et al. [173], it is expected that chemical cross-linking restricts conformational changes induced by heat and stabilizes the structure of the enzyme, increasing the stability of macromolecular cross-linked aggregates, as shown in their work.

Co-aggregation of enzymes and other proteins rich in lysine residues, such as bovine serum albumin (BSA), increase the concentration of highly reactive amino groups, facilitating the formation of the stable CLEAs [48,174]. Torabizadeh et al. [174] introduced a two-step method for the preparation of CLEAs of a thermostable α-amylase, including calcium and sodium ions during the enzyme aggregation. The authors selected the best ratio of enzyme:BSA, obtaining 6.2% higher conversion than CLEAs without BSA addition. There was no significant change in the optimum pH of the enzyme activity after immobilization, but the activity and stability of produced CLEAs were significantly increased in the presence of calcium and sodium ions.

Talekar et al. [163] recovered 45% of applied α-amylase activity in CLEAs and 100% when magnetic CLEAs were used, this activity remaining unchanged even after 100 batch reuses. This high-recovered activity was achieved by adding amino functionalized magnetite nanoparticles into α-amylase solution, increasing the amine groups available to cross-link with glutaraldehyde. Moreover, the temperature for the highest α-amylase activity was established at 45 °C for the free enzyme, shifting to 50 °C for CLEAs, and to 60 °C for magnetic CLEAs. The authors assumed that this increased thermal protection is due to the higher amount of covalent cross-linking between enzyme and amino-functionalized magnetite nanoparticles in magnetic CLEAs compared to CLEAs without addition of external amino groups. Improved thermal stability, storage stability, and reusability of magnetic CLEAs is an attractive way towards stable CLEAs preparation, and could overcome the drawback of CLEAs clumping.

The enzymatic process of starch hydrolysis and sugars production involves the use of different enzymes, besides *α*-amylase: glucoamylase, an exo-acting enzyme that hydrolyzes α(1-4) and α(1-6) glycosidic bonds from the non-reducing ends, and pullulanase, which differs from amylases because it hydrolyzes pullulan, in addition to amylopectin. In the presence of high concentrations of glucose and dissolved solids, glucoamylases can also catalyze reverse condensation reaction decreasing overall glucose yield. The addition of a pullulanase, a debranching enzyme which has also the capability to hydrolyze α(1-6) glycosidic bonds, can be an alternative to improving the glucoamylase performance at this conditions. [154,175,176]. In this way, Talekar et al. [177] developed a carrier free co-immobilization of these three enzymes aiming at using this combi-biocatalyst in one batch reaction. The authors reported 100% of starch conversion with this combi-biocatalyst, compared to only 60% and 40% when single CLEAs mixture and free enzymes were used, respectively.

- Other approaches for α-amylase immobilization:

Magnetic poly(2-hydroxyethylmethacrylate) beads carrying a dye-ligand (Cibacrom Blue) for specific proteins ligand was used for α-amylase immobilization by adsorption with a load of 401 ± 11 mg/g support. After adsorption, the optimal pH shifted from 7.0 to 8.0, and the pH profile of the immobilized α-amylase was much broader than that for the free enzyme, indicating a promoted protection by immobilization. This protection was also able to preserve the enzyme from temperature damage, showing a maximal catalytic activity at 10 °C higher than the free enzyme [178]. Guo et al. [160], used magnetic Fe_3_O_4_ nanoparticles functionalized with 3-aminopropy-ltriethoxysilane (APTES) for the immobilization of porcine pancreatic α-amylase and reported that the enzyme exhibited higher temperature and pH resistance, in addition of better organic solvent tolerance. The authors argued that the immobilization process enhanced the rigidity and decreased thermal perturbations of the enzyme structure. Magnetic nanoparticles were coated with gum acacia and, using glutaraldehyde, they formed covalent bonds with α-amylase, enabling higher immobilization yields (60%) than when using unmodified magnetite nanoparticles [159].

Microspheres of gellan gum, a linear polysaccharide formed by units of glucose, glucuronic acid, and rhamnose was used to entrap α-amylase through ion tropic technique. The relative activity in the presence of amylopectin, maltodextrins, and glycogen was 73%, 85%, and 14%, respectively. It was possible to inversely correlate enzyme activity with the size of the substrate [158]. Gashtasbi et al. [179], designed a new approach, using adsorption and covalent methods, to immobilize *B. licheniformis* α-amylase on the surface of *B. subtilis* spores. For the covalent method, 1-ethyl-3-(3-dimethylaminopropyl) carbodiimide (EDC) and sulfo- N- hydroxysuccinimide (NHS) were used to activate free carboxyl groups on the spore EDC surface, followed by a condensation reaction with the amino groups of the enzyme. The optimum pH of immobilized α-amylase shifted from 5.0 to 8.0, an expected behavior because of the anionic surface of the spore. The covalently immobilized enzyme was stabler at higher temperatures, and its maximum activity changed from 60 °C to 80 °C. This method also allowed the enzyme to retain 64% of its initial activity after 10 reaction cycles.

Ionic exchange and hydrophobic interactions were tested for α-amylase immobilization on gold nanorods. The immobilization promoted significant enhancement in the thermal and pH stabilities compared with the free enzyme. The immobilized enzyme showed higher activities, both in acidic and basic pH ranges, compared to the soluble form, exhibiting 33% of activity at pH 2.0 and 52% at pH 10, whereas the soluble enzyme was almost fully inactivated under the same conditions. Concerning temperature, maxima activities of free and immobilized α-amylase were observed at 50 °C and 60 °C, respectively, whereas their irreversible thermal inactivation were determined to be 70 and 80 °C, respectively. However, the immobilized enzyme presented a thermal protection, since its half-life at 80 °C, was three times higher than for the free form. Moreover, the immobilization allowed the retention of 45% and 20% of the original activities after 60 min of incubation at 70 and 80 °C, respectively. The slightly decrease of k_cat_ and increase of K_m_ may be explained by enzyme structure distortions associated with the immobilization [180].

### 4.2. Cyclodextrin Glycosyltransferase

Cyclodextrin glycosyltransferases (CGTase, EC 2.4.1.19) are other important members of GH13 family of enzymes [181,182]. As for α-amylases, CGTases use the so-called *α*-retaining double displacement mechanism to react with starch substrates [183]. These enzymes can be produced by a variety of bacteria, especially of the genus *Bacillus* [184,185,186], having molecular weights around 75 kDa and five domains—labeled A to E. The domain A contains the catalytic (α/β)_8_ domain, which characterizes the members of this family [149,187].

Besides the hydrolysis reaction, CGTases catalyze three inter- and intramolecular transglycosylation reactions: disproportionation, coupling, and cyclization, always acting on α-(1,4) glycosidic bonds. The cyclization reaction has been of great interest, since this is the unique enzyme that can produce the cyclic α-1,4-glucan with 6, 7, and 8 degrees of polymerization, named as α-, β-, and γ-cyclodextrin [188,189,190,191]. Cyclodextrins (CDs) have a hydrophobic central cavity that can incorporate various inorganic and organic compounds, forming inclusion complexes with them [192]. Therefore, these CGTase products are widely used in the pharmaceutical, food, agricultural, and cosmetic industries [186,193,194,195,196,197].

All native CGTases produce mixtures of α-, β-, and γ-CDs and the isolation and purification of a specific CD is a rather expensive and time-consuming process. According to Biwer et al. [186], solvent process purification can only isolate the CDs from the rest of the reaction mixture, but not the different CDs types from each other. Since each of them has a dimensionally distinct central cavity and different specificity for guest molecules [198,199,200], studies have focused on to find or engineer a CGTase that produces a specific type of CD. Additionally, it is clear the necessity for novel CGTases with improved properties related to their stability, and developments to better modulate the reaction for the production of a specific CD [149,188].

According to Kelly et al. [201], the type of cyclodextrin produced by CGTases is dependent upon the number of glucose units that bounds at the donor substrate binding site prior to the glycosidic bond cleavage. There is an evolutionary diversification among CGTases regarding both cyclodextrin product specificity and thermal stability property. Furthermore, mutations (substitution, insertion, and/or deletion) in one or more of conserved residues at the donor subsites can directly affect the product specificity of these enzymes. Considering the importance of central tyrosine 195 in the active site of CGTase, Xie et al. [202] applied site-directed saturation mutagenesis to investigate its role on the hydrolytic and cyclization specificity of an *α*-CGTase. Interestingly, the authors found mutants that drastically altered the CD specificity from initial 35% of α-CD to 34% of β- and 38% of γ-CD (Y195I) or even to 50% of γ-CD (Y195R).

#### 4.2.1. Immobilization of CGTases

Immobilization of CGTases has been developed in the attempt to reduce the costs of cyclodextrins production. Several approaches have been applied, such as adsorption [203], entrapment [204], and covalent binding [205].

Different combinations of supports and methods were studied by Sobral et al. [206] to immobilize CGTase from *B. firmus*. Adsorption and covalent immobilizations were tested using silica, chitosan, and alumina supports. The highest activity of immobilized enzyme was obtained for the covalent immobilization in chitosan. Polyethylene film functionalized with carboxylic acid groups was used as support for immobilization via covalent bonds with carbodiimide (EDC). The CGTase from *B. macerans* showed an increased production of the three types of cyclodextrins, according to the reaction time, following the order: α-CD > β-CD > γ-CD [207]. CGTase produced by *Paenibacillus macerans* was immobilized on aminated polyvinylchloride (PVC) by covalent binding with glutaraldehyde, having its thermal stability and resistance to chemical denaturation improved. Both free and immobilized enzymes had an optimum pH of 6.0, but the immobilized presented higher activities at lower pH values. The free enzyme showed optimum activities at temperature of 60 °C, whereas for the immobilized form optimum temperature was 75 °C [208].

The processing of starch requires the use of high temperatures to obtain its liquefaction, usually using *α*-amylase, thus it would be of interest to find thermostable CGTases that could be used in these processes. Norman and Jorgensen [209], isolated a thermostable CGTase from *Thermoanaerobacter* sp. ATCC53627. This is an extremely heat-stable enzyme, with optimum temperature activities of 90 to 95 °C at pH 6.0, and, in the presence of starch, it is even stable above 100 °C. Therefore, liquefaction and cyclization may be carried out without further enzyme addition, yielding products with high purity grade. The gene encoding this *Thermoanaerobacter* CGTase was cloned into a *Bacillus*, and this enzyme is now commercialized by Novozymes A/S as Toruzyme 3.0L, and has been used in several studies.

Tardioli et al. [210] tested the multipoint covalent immobilization of CGTase in cross-linked 6% agarose beads activated with aldehyde groups (glyoxyl-agarose). The authors obtained increased rigidity of the CGTase (Toruzyme 3.0L), thus a higher resistance to conformational changes caused by denaturing conditions, such as pH and temperature. Satisfactory results were obtained, as higher operational stability at 85 °C, 30% of the substrate conversion with only 2 h of reaction, and a two-fold increase in production rate compared to the free enzyme. Covalent attachment of Toruzyme CGTase was also carried out using Eupergit C as support. This support contains oxirane (epoxy) groups instead of aldehyde groups, to attach the proteins. Although it was not observed any change in the optimum pH of the immobilized enzyme, it was identified a broader pH range with high catalytic activity, especially at high pH values, and the optimum temperature changed from 85 °C to 80 °C [205]. The immobilization on glutaraldehyde-activated chitosan spheres changed the optimal temperature of β-CGTase activity from 75 °C to 85 °C at pH 6.0. Free and immobilized enzymes presented optimum pH at 5.0, but the immobilized-chitosan particles presented higher operational stability, with 61% of its initial activity still remaining after 100 batches [211]. The same optimum pH was observed when mesoporous silica functionalized using 3-aminopropyltrimethoxysilane (APTMS) was used to immobilize the enzyme, but the best temperature for β-CD production changed from 100 °C to 80 °C after immobilization [212].

Although some studies have revealed good immobilization efficiencies (73% with macroporous silica [212], 32% with glyoxyl-agarose [210], 47% and 25% for ionic-exchange resins [213]), in general, it has been reported low activities recoveries of CGTases after immobilization. These activities were in the range of 5.4% on Eupergit C [205], 6.1% on chitosan by covalent method [211], and 3.6% by adsorption [203]. These reductions in specific activities after immobilization could be caused by steric hindrances, internal diffusional limitations, modifications of the enzyme conformation and/or the active site, as well as to microenvironment of the support matrix, which can differ significantly from the natural environment of the enzyme [205]. However, the possibility of reuse of the biocatalyst can overcome these drawbacks, because good operational stabilities after the immobilization have been observed. For instance, immobilized CGTases presented 40% of its initial catalytic activity after 10 cycles of 24 h when attached to Eupergit C [205], 60% after 100 reuses when on chitosan [211], 85% after 14 cycles on PVC [208], and about 60% after 15 cycles on mesoporous silica [212].

The availability of extremely stable CGTases encouraged studies for their utilization in continuous reactors for the production of CDs [214]. For instance, Schöffer et al. [211] evaluated the operational stability of a thermostable CGTase immobilized on spheres of chitosan in a packed-bed reactor. The results showed that 100% of the initial activity was observed even after 100 h of continuous use, indicating a promising option for its use in an industrial process. Tardioli et al. [190], tested a fluidized-bed reactor using immobilized CGTase onto silica particles and produced, in only 4 min of residence time, the same amount of CDs normally achieved using the free enzyme after 24 h in a batch reactor. Under the reaction conditions used by the authors, it was possible to obtain a selectivity of 82% of β-CD [215]. The use of continuous reactors has several advantages, because it simplifies the purification process of products and allows for a more rational approach of scaling-up [37].

Studies involving genetic engineering have gained more attention in the last decade for the cloning of CGTase genes. For instance, the gene coding for CGTase of *B. macerans* was fused to consecutive 10 lysine residues; its product was electrostatically immobilized on a cationic exchanger by adsorption. This new CGTase showed high thermal and operational stabilities. The operational half-life of this poly-Lys enzyme in a packed-bed reactor was 12 days at 25 °C and pH 6.0 [216]. This modified biocatalyst also presented the advantage of enabling directional immobilization on the solid surface without blocking the active site [217].

## 5. The GH 70 Family of Enzymes

### 5.1. GH 70 Enzymes and Their Characteristics

Glucansucrases, also known as glucosyltransferases, are extracellular enzymes classified as members of GH70 family, based on four catalytically conserved sequence motifs, which are similar to those of the families GH13 and GH77 [218,219]. The GH70 family comprises dextransucrase, mutansucrase, alternansucrase, reuteransucrase, and α-4,6-glucanotransferase, enzymes having high molecular weight, in the range of 120–200 kDa. These enzymes are produced by several lactic acid bacteria strains, such as *Leuconostoc*, *Streptococcus*, *Lactobacillus,* and *Weisella*, microorganisms that are Generally Recognized as Safe (GRAS) [220]. 

Regarding the structure of glucansucrase, its catalytic domain is predicted to be organized in a (α/β)_8_-barrel resembling those of GH1 and GH13 enzyme families, but probably circularly permuted. Sequence comparisons between the GH13 and GH70 families enabled the identification of only one catalytic triad composed of two aspartic acids and one glutamic acid: Asp-551, as nucleophile, Glu-589, as the acid-base catalyst, and Asp-662, as assistant in the glucosyl-enzyme formation [221]. These residues are strictly conserved for all the GH70 family enzymes and their mutations lead to enzyme inactivation [218,222,223]. Furthermore, it has been reported that there are some amino acids, such as lysine, present in the catalytic domain of dextransucrase, which are responsible for anchoring the substrate and elongating the oligosaccharides in the acceptor reaction [224,225,226]. The modification of these amino acids is related to enzyme denaturation, probably caused by conformational changes [224,225].

The amino acid sequence of dextransucrase includes a signal peptide, followed by a variable stretch of approximately 200 amino acids, a conserved core region of about 900 amino acids (N-terminal catalytic core domain), and a series of direct repeating units of about 400 amino acids (C-terminal glucan-binding domain) [227]. This last domain is related to the interaction with sugars during the reaction and possibly aid the anchoring of the growing polymer to the enzyme surface [228].

The glucansucrases catalyze four types of reaction: (i) polymerization, in which occurs the transfer of glucosyl moieties in presence of sucrose onto α-glucans, with the release of fructose as a by-product [229,230]; (ii) hydrolysis, in which water is used as an acceptor substrate; (iii) acceptor reaction, when glucosyl moieties are transferred to the non-reducing end of acceptor molecules to produce oligosaccharides [218]; and (iv) disproportionation reaction, which is the transfer of the non-reducing end of an α-glucan chain to another α-glucan chain. Particularly, α-4,6-glucanotransferase only catalyzes the disproportionation reaction [231].

The products of polymerization reactions can differ in their glycosidic linkages. Each reaction product may contain a specific pattern: dextran has mainly α-1,6-bonds; mutan, mainly α-1,3-bonds; alternan has α-1,3- and α-1,6-bonds; and reuteran, mainly α-1,4-bonds [232]. Similarly, the products of acceptor reactions can vary in terms of linkages types, size, degree of branches, and spatial arrangements [228]. Maltose, glucose, fructose, d-mannose, and cellobiose have already been evaluated as acceptors for glucansucrases reactions, resulting in different oligosaccharides, such as gluco-oligosaccharides, isomalto-oligosaccharides, among others [233,234,235,236,237,238].

In this review, we will focus in a specific class of glucansucrases, the dextransucrases (E.C. 2.4.1.5). The bacteria *L. mesenteroides* NRRL B-512F, *L. mesenteroides* B-512FMCM, *L. mesenteroides* NRRL B-1299, and *L. citreum* KACC 91348P are among the main microorganisms that synthetize this GH70 enzyme [239,240,241,242]. As stated above, dextransucrase catalyzes the synthesis of dextran and oligosaccharides. The dextran produced in the reaction is tightly bound to the protein, which promotes stability to the biocatalyst [218,243,244].

There is growing interest in the enzymatic synthesis of dextrans and oligosaccharides because these products find extensive use in the food, feed, cosmetic, and pharmaceutical industries [245,246]. Dextrans have beneficial properties to human health due to antitumoral, immunomodulatory, cholesterol-lowering, and biofilm-formation-inhibiting activities [247]. As functional ingredients, these polysaccharides are versatile across a broad range of applications in the food industry as viscosifying, stabilizing, emulsifying and gelling agents [248]. Additionally, the oligosaccharides have prebiotic potential. An ingredient is classified as prebiotic when it is not digested in the stomach and it is selectively digested by *Bifidobacteria* and *Lactobacillus* in the intestine, stimulating the growth of these beneficial bacteria and, as consequence, improving the host health [249,250,251,252]. Dextransucrases have been used to convert the high sugar concentration present in beverages, like orange, mandarin, and cashew apple juices, into prebiotic oligosaccharides [237,253,254].

### 5.2. Immobilization of Dextransucrase

For the industrial application of dextransucrase, either for the production of dextrans or oligosaccharides, an effective immobilization technique is required to assure continuous processing and reuse of the biocatalyst. Moreover, the immobilization process can promote innumerous other advantages, as mentioned before for the other enzymes in this review.

#### 5.2.1. Immobilization of Dextransucrase by Entrapment

In the literature, the most studied technique for dextransucrase immobilization is the entrapment or encapsulation. This method was used by different groups and seems to be the most convenient, because the entrapment enables the immobilization without chemical linkages between the enzyme and the support. Compared to other strategies, the encapsulation presents higher immobilization yields, varying from 57% to 98% [255,256,257,258,259]. The enzyme-dextran complex has higher molecular weight, then the dextran-free enzyme, possibly resulting in a more suitable molecule to be retained into the gels [255].

This immobilization protocol presents some advantages and disadvantages. The major problem of this technique is the internal diffusion restriction in the beads. The high dextran content surrounding the dextransucrase probably causes a decrease in the enzymatic activity because of mass transfer phenomena limitation, as it has been reported by Berensmeier, Ergezinger, Bohnet and Buchholz [244]. As consequence, the alginate-immobilized dextransucrase can only be used for oligosaccharides production, since the high molecular weight of dextran cannot diffuse out of the beads without their rupture [260,261]. This polymer layer is also the reason for limited operational stability of the biocatalyst and its lower productivity when it is used in continuous operation. Beyond that, the enzyme can be leaked out of the beads, as result of swelling, and their remaining enzymatic activity is considerably reduced [255,262,263].

Comparing dextransucrases from different strains such as from *L. mesenteroides* B-512 F and *L. mesenteroides* B-1299, it is clear that these enzymes demonstrate unique characteristics. While B-512 F dextransucrase showed recovered activity of 84% after entrapment in calcium alginate, compared to only 57% of the B-1299 dextransucrase [263]. The distortion caused in the beads are also different. For the immobilized dextransucrase B-1299, the major product is the oligosaccharide with α-1,2 bonds, whereas immobilized B-512 F dextransucrase produces dextrans. These singular products affect the bead conformation, since dextran remains inside the particles, whereas the prebiotic sugars are released to the medium, consequently maintaining the spherical shape of the beads [264].

Diffusion problems in encapsulation-immobilized dextransucrase may also be influenced by the content of dextran, and the size and shape of the particle. It has been observed that only 5% of dextran is surrounding the enzyme, presumably covalently bonded, which can promote and stabilize the dextransucrase immobilization [258,263]. Concerning the particle size, an optimal diameter of the alginate beads, which varies from 1 to 5 mm, allows the correct diffusion of substrate and products [244,255,258]. Concerning this aspect, Tanriseven and Doǧan [235] comparing the shape of alginate fibers and beads, reported immobilization yields of 90% and 60%, respectively [260].

Another advantage of dextransucrase encapsulation relates to the simplification of the segregation of products, namely dextran and oligosaccharides, since dextran is limited to the bead microenvironment owing to its molecular size, whereas the smaller oligosaccharides are released to the reaction medium. Furthermore, the alginate matrix possibly acts as a protection for the biocatalyst, with the half-life of the entrapped dextransucrase, which is always reported as being higher than that of the free enzyme [244,258,262]. Moreover, glucose and maltose could be added as additional stabilizers [256,265], and soluble starch as viscosity modulator, protecting the enzyme [266]. In continuous processes, this stabilization effect is even more important, especially within reaction conditions, as evaluated for the production of isomaltose and gluco-oligosaccharides [256,259]. For instance, Berensmeier et al. [219] compared the stability of calcium alginate immobilized and free dextransucrase by measuring their half-life at 30 °C and reported that the immobilized enzyme had this property reduced from 23 h to 0.6 h when the dextran layer was removed by dextranase treatment. The encapsulation may also promote different product selectivity, which is a very interesting property of the system. Although this finding is difficult to explain, it has been suggested that is probably related to the nature of the support and its interaction with the enzyme [267,268].

Stable preparations of immobilized dextransucrase may also be obtained by cross-linking the enzyme with glutaraldehyde prior to the entrapment in alginate beads and by coating these beads with chitosan films. Kubik, et al. [269] found high operational stability for immobilized dextransucrase cross-linked with 10% of glutaraldehyde. The immobilized biocatalyst could be used for 10 reaction cycles for isomalto-oligosaccharides production, reaching 60% to 70% of sucrose conversion, even in the last batch. When chitosan was used to coat the alginate beads aiming to promote stabilization of particles, the sucrose conversion and the isomaltose yields were slightly reduced, probably caused by diffusional limitations. Nevertheless, the chitosan, linked via electrostatic interactions with the alginate beads, avoided the leakage of encapsulated enzyme to the reaction medium [270]. These findings also demonstrate that the enzyme entrapped in alginate, without cross-linking, would leak out of the support, especially when dextranase is applied in the reaction medium.

The calcium alginate immobilized enzyme has been evaluated in fluidized and packed-bed reactors [262,271], and depending on the reactor configuration, some variables may be studied in order to optimize the process. Packed-bed reactor was used to continuously synthesize gluco-oligosaccharides (GOS) and it was observed the accumulation of dextran inside the beads, probably because of the high amount of initial sucrose concentration [262]. This microenviroment—forming a bead/enzyme-dextran complex—can be related to the swelling of beads. Quirasco, Remaud-Simeon, Monsan and López-Munguía [271] demonstrated that the whole cell from *L. mesenteroides* B-1299 could be encapsulated in alginate beads in order to produce GOS in a packed-bed reactor, with immobilization yields of 93%. These authors reported similar problems for encapsulated whole cells as reported for the purified biocatalyst.

Berensmeier et al. [219] designed a fluidized-bed reactor to operate with a high-density fluid phase of concentrated sugar solutions to produce isomalto-oligosaccharides. In order to overcome the possibility of flotation of the beads, the authors used silica flour added to alginate to increase the bead density [244]. Another important point is the weakly repressed dextran formation. It has been demonstrated that it is possible to obtain higher mechanical stability of the immobilized catalyst and higher oligosaccharides yields with the optimal sucrose/acceptor ratio in the reaction. For long-term operation, sucrose should be kept at low concentrations, whereas the acceptor concentration should be high to avoid dextran synthesis [244,256]. Some authors reported that sucrose in high concentrations might block the reaction chain of oligosaccharides because some sugar molecules are linked to the enzyme in its allosteric site affecting the shape of the protein. Therefore, the glucose residues anchored in the dextransucrase active site cannot form the glycosidic bond [272].

Besides dextransucrase encapsulation, the co-immobilization of this enzyme with dextranase (EC 3.2.1.11) is another way to efficiently synthesize dextrans and oligosaccharides [238,273]. This technology allows the design of novel multi-functional biocatalysts and can display benefits owing to the synergy of the enzymes [274,275]. However, the challenge of this kind of co-immobilization is to avoid the inactivation of dextransucrase that is promoted by dextranase. The enzyme-dextran complex can be cleaved up to 97%. Because endogenous dextran is essential for the retention of dextransucrase activity, the enzyme stability is related to the presence of a dextran layer [244]. At the same time, the hydrolytic activity of dextranase may regulate the molecular size of the product and the availability of the acceptor, in such a way that the synthesis of products can be directed to obtain desirable characteristics, such as prebiotic effect [276].

Erhardt, Kügler, Chakravarthula and Jördening [238] studied the immobilization of dextranase prior to co-entrapment with dextransucrase into calcium alginate. Hydroxyapatite was found to be the best support, since it was almost inert to dextransucrase and was ideal to adsorb dextranase. Their findings suggest that the co-immobilization on a solid phase prior to entrapment suppresses bead swelling owing to reduced dextran formation and slows dextransucrase inactivation. The co-immobilized biocatalyst kept only 25% of its initial activity after 6 batches, when the ratio of dextranase/dextransucrase activities of 0.3:1 was used.

Ölçer and Tanriseven [266] developed a simple and effective co-immobilization method of dextransucrase/dextranase bearing potential for industrial-scale production of isomalto-oligosaccharides. These compounds can be produced either by acceptor reactions of dextransucrase or hydrolysis of dextran by dextranase as already reported by Goulas, Fisher, Grimble, Grandison and Rastall [276]. An important aspect of this particular immobilization was the pre-immobilization of dextranase by covalent attachment on Eupergit C prior to alginate co-immobilization (beads, fibers, and capsules), preventing dextranase leakage and dextransucrase inactivation. The best immobilization yields was 71% in alginate capsules. The enzymes retained their activities during 20 repeated batch reactions and for a month when stored at 4 °C [266]. Goulas et al. [273,276] investigated the production of isomalto-oligosaccharides using the free form of both enzymes, in batch and in a continuous recycling ultrafiltration membrane reactor. The authors reported similar yields of sucrose conversion by using free dextransucrase or its immobilized preparations. However, when the authors co-immobilized the dextransucrase and dextranase, the rate of formation and the size of acceptor products were regulated, in contrast with the use of absolute concentrations, leading to polymers with more persistent prebiotic effect.

Another interesting material for the entrapment of biocatalysts is the hydrogel formed by polyvinyl alcohol (PVA). PVA is highly elastic, stable, and suitable as entrapper to immobilize dextransucrase. Commercially available PVA-particles (LentiKats^®^) have lens-shaped form, a diameter in between 3 and 5 mm, and a thickness of 300 to 400 mm, and they have been used to immobilize dextransucrases. Dextransucrase from *L. mesenteroides* B-1299 encapsulated in LentiKats^®^ had similar recovery of activity (approximately 55%) when compared with entrapment in calcium alginate gels. In addition, the conversion to α-1,2-linear and branched oligosaccharides using LentiKats^®^-dextransucrase was higher than that obtained for alginate-dextransucrase, probably because of the reduction of diffusional limitations derived from its lenticular shape. Other important parameter is the protein leaching. This problem was reduced from 18% to 4% by pre-treating dextransucrase with glutaraldehyde. However, this complex glutaraldehyde-enzyme possibly affected some amino acids in the catalytic site, as the yields and the specific activity of this preparation were lower than expected [268].

#### 5.2.2. Immobilization of Dextransucrase by Covalent Immobilization

Several groups have reported the covalent immobilization of dextransucrase. In this case, the carriers must display high density of reactive groups for attachment of the enzyme to the support. The enzyme was covalently immobilized in Bio-Gel P-2 [277], polyacrylamide gel, cellulose acetate membranes, polysulfone hollow fiber [278,279], and akylamine porous silica [280]. The covalent binding of dextransucrase to porous silica activated with α-aminopropyl and glutaraldehyde has also been described [265,281]. Most of these studies reported low immobilization yields, low specific activities and poor operational stability. These findings indicate that some of the reactive groups present at the catalytic domain, such as lysine, could react with the aldehyde and epoxy groups of the covalent immobilization matrices. Moreover, the dextran associated with the enzyme covers the reactive groups on its surface, which possibly affects the covalent immobilization of dextransucrase [224,261,282].

Epoxy supports have been demonstrated to be suitable supports for enzyme immobilization, bearing industrial potential [283,284]. These epoxy-activated supports were able to chemically react with different nucleophile groups placed on the protein surface: lysine, histidine, cysteine, tyrosine, among others [285]. Gómez de Segura, Alcalde, Yates, Rojas-Cervantes, López-Cortés, Ballesteros and Plou [263] immobilized the dextransucrase on epoxy activated acrylic polymers with different textural properties (Eupergit C and Eupergit C 250L). In order to promote the accessibility of reactive groups of the enzyme surface to the epoxide centers of the support, easing the covalent coupling, the native dextransucrase, which may contain up to 80 g glucose/g protein [286], was treated with dextranase to assure the removal of dextran layer. These findings suggest that Eupergit C 250L works better to bind enzyme molecules inside its macroporous matrix, since has higher volume and higher diameter of pores compared to Eupergit C, which explains its higher specific activity (up to 710 U/g). The maximum activity recovered was 22%, with an immobilization yields of 72% using Eupergit C 250L and the immobilized enzyme kept more than 40% of its initial activity over 2 days at 30 °C and pH 5.4. Unlike alginate beads, the authors reported that dextransucrase did not diffuse into swelled matrices [263].

Hashem, et al. [287] evaluated the covalent immobilization of dextransucrase from *Enterococcus faecalis* Esawy by Fe^3+^-cross-linked alginate/carboxymethyl cellulose beads modified with polyethylenimine and glutaraldehyde. The immobilization yields reached 94.35%. Moreover, the immobilization process improved the thermal and pH stability of the enzyme to great extent, probably caused by covalent attachment that protects against protein conformational changes [41,285]. Reusability tests proved that the enzyme retained 60% of its initial activity after 15 batch reactions [287].

Alcalde, Plou, De Gómez Segura, Remaud-Simeon, Willemot, Monsan and Ballesteros [258] investigated the immobilization of dextransucrase by covalent attachment on activated silica (silica X030). The removal of the dextran covering the enzyme surface was essential to promote the bonding and, indeed, while the dextran-free enzyme showed immobilization yields of 13%, the native dextransucrase presented yields of only 0.6%. These low immobilization yields might be related to the participation of a lysine residue in the catalytic domain of dextransucrase. The interaction of this Lys with the support probably changes the protein conformation, reducing its catalytic activity [224,288,289]. The residual activity of the biocatalyst immobilized on the silica decreased from 58% to 17% after 48 h of reaction, fact that did not occur when the dextransucrase was immobilized in alginate beads [258].

#### 5.2.3. Immobilization of Dextransucrase by Adsorption

There are few works on dextransucrase immobilization using the methodology of adsorption. Kaboli and Reilly [280] tried to attach the dextransucrase from *L. mesenteroides* B-512 F by anion exchange to DEAE-cellulose, DEAE-Sephadex A-25 and A-50, and by cation exchange SP-Sephadex C-25 and C-50, without satisfactory results. Hydroxyapatite [290], Sephadex G200 [291], and phenoxyacetyl cellulose [292] have also been studied as carriers to adsorb the biocatalyst, all of them without success.

#### 5.2.4. New Approaches in Dextransucrase Immobilization

Some research groups have been using different protein engineering tools to enhance several dextransucrase parameters, such as the catalytic activity and type of reaction products [223,228,293]. These strategies, combined with immobilization techniques, have provided good results. Parlak, Ustek and Tanriseven [261] developed a bioengineering study, in which a novel dextransucrase was fused to a glutathione-S-transferase (GST), to facilitate the covalent immobilization on Eupergit C 250L. The modification provided additional 21 lysines, 18 aspartic acids, 16 glutamic acids and 4 cysteines. The results showed the importance of the fusion protein because the immobilization yields and recovered activity were 100% and 83.3%, respectively. Comparatively, under the optima conditions, the immobilization of truncated dextransucrase without GST resulted in 100% of immobilization yields, but only 3% of recovered activity. Furthermore, the immobilized enzyme showed no decrease in activity for 15 batch reactions and retained its initial activity at 4 °C storage for 35 days.

The challenge remains about the glucansucrase structure and its surface reactive groups to improve the immobilization parameters. Until now, there are only five three-dimensional structures known of GH70 glucansucrases: glucansucrase GTF180-ΔN from *L. reuteri* (PDB: 3KLK; [294]), glucansucrase from *S. mutans* (PDB: 3AIE; [295]), dextransucrase DSR-E from *L. mesenteroides* NRRL B-1299 (PDB: 3TTQ; [296]), glucansucrase GTFA from *L. reuteri* 121 (PDB: 4AMC; [297]), and dextransucrase DSR-E from *L. mesenteroides* NRRL B-1299 (PDB: 4TVD; [298]). Comparatively, the Protein Data Bank (www.rcsb.org) presents more than 200 three-dimensional structures of members of GH1 and GH13 families. This lack of information about the crystal structures of glucansucrases makes difficult to predict which bonds will be involved in the immobilization process.

Further developments in elucidating aspects of the domains of glucansucrases must also be addressed. The glucan-binding domain, found at C-terminal, is a highly conserved region. Some authors discovered that this domain plays a major role in polymer elongation, since it is a sucrose and/or polymer-binding site [224,299] and its truncation can result in an enzyme much less efficient in catalyzing high molecular weight polymers [228]. On the other hand, this domain does not seem to be involved in linkage specificity [228], and it has been demonstrated that the truncation of dextransucrase in this region, associated with the fusion to other protein, preserves the catalytic activity and enables the immobilization with satisfactory results [261]. Therefore, it is important to find which domains could interact with supports, and to evaluate if these bonds are responsible for activity losses or enzyme inactivation.

Kaboli and Reilly [280] stated in 1980 that “dextransucrase is an extraordinary difficult enzyme to immobilize”. In fact, it has been demonstrated that glucansucrases present some shortcomings. To the best of our knowledge, there is no work with glucansucrases immobilized via adsorption or covalent binding applied in continuous reactors. Therefore, there are still many aspects to be investigated about their immobilization and their behavior under continuous operation applied to synthesize different desirable products. Further research is in need to develop a stable, versatile, and robust immobilized biocatalyst based on glucansucrases.

## 6. Future Perspectives

We discussed the main protocols used to immobilize the enzymes from GH1, GH13, and GH70 families of enzymes, with special focus on β-glucosidases, α-amylases, cyclodextrin glycosyltransferase, and dextransucrase. Immobilization is not only a strategy to turn the soluble enzymes into heterogeneous catalysts, but it is also a way to improve and/or change their activities, stabilities or specificities. In general, developments in material science and molecular biology are the main tools to obtain new support matrices and to improve enzyme properties for immobilization.

In the case of the GH1 family, although carriers and immobilization methods have been intensively investigated, there are still few studies using techniques as co-immobilization on solid supports, or the preparation of CLEAs, or even combi-CLEAs, using the β-glucosidase in combination with other enzymes. These studies are very interesting because the co-immobilization of several enzymes can be applied in sequential biocatalytic processes, thus reducing costs for industrial applications. In addition, recent progress on the field of the structural characteristics of β-glucosidases from GH1 family, have been providing the starting point to improve and orientate the immobilization by site-directed mutagenesis on enzyme surface following the immobilization on solid supports. This may increase the stability and could be useful to obtain mutants with more advantageous characteristics, such as lower product inhibition, and higher activities and/or specificities.

Concerning the GH13 family, although CGTases are well known for their use in the production of CDs, many additional applications have been explored based on the other reactions catalyzed and a huge industrial demand for these enzymes has emerged over the last decades. Efforts have been made to improve these enzymes to better suit industrial applications by immobilization techniques. Recent researches are being directed to improve the enzyme performance using the heterologous expression and molecular engineering. Site-directed mutagenesis has been applied extensively, enhancing properties such as substrate conversion, product specificity, stability, and specific activities. However, further developments are still necessary in order to biological models suitable for the heterologous expression aiming at improving the quantity and quality of these enzymes and to enable their use in different areas.

Glucansucrases from GH70 family were first immobilized more than 40 years ago. However, a high-recovered activity of immobilized biocatalyst for industrial applications remains as a challenge. So far, best technologies for the immobilization of glucansucrases are mainly based on entrapment. Nevertheless, as for β-glucosidases, CLEAs appear as a new perspective to be used with glucansucrases. Finally, further investigations on protein engineering combined with immobilization techniques may be useful to identify amino acids that can be modified in order to enhance the immobilization parameters of glucansucrases.

## Figures and Tables

**Table 1:** Characteristics of different covalent binding based immobilizations of β-glucosidases.

Reactive Group	Immobilization Support	Reference
Glutaraldehyde	pore glass particles	[81]
	chitosan and alginate beads	[70,76]
	Bentonite, celite, silica gel, and Nylon	[82]
	mesoporous silica MCM-41	[83]
	SiO_2_ nanoparticles	[84]
	Chitin, Loofa, Sawdust coarse, Sawdust fine, Sponge, Stainless steel, Pumice, Wool and agar, agarose and sodium alginate	[72]
	Chitopearl beads BCW-3001	[85]
	amine agarose gel	[86]
	silica gel	[87]
	nylon powder	[88]
	chitosan–clay composite	[78]
	agarose matrix	[89]
	chitosan	[74,75,77]
	magnetic chitosan microspheres	[80]
	chitosan-carbon beads	[79]
	Spent coffee grounds	[90]
	iron oxide magnetic nanoparticle	[91]
	polyvinylalcohol (PVA) nanofibrous membranes	[92]
	mesocellular silica foams	[93]
l-lysine and glutaraldehyde	chitosan microspheres	[94]
Hexamethylenediamine and glutaraldehyde	chitin (IME-C) and calcium alginate (IME-A)	[95]
Polyelectrolytes (PEI) and glutaraldehyde	Kappa-carrageenan beads	[96]
APTMS and glutaraldehyde	cellulose PEI, alpha-alumina, gamma-alumina and chitosan	[97]
Epoxy	Eupergit C 250L	[98]
	polyacrylic matrices supports (Eupergit^®^ C, Eupergit^®^ C250L, and cryogel)	[99]
	Eupergit C	[100]
Nylon-hydrazide	nylon pellets	[101]
CNBr	sepharose gel beads 4B	[102]
Carbodiimide	magnetic beads	[103]
Mercaptopropyl-functionalized	Mesoporous titanium dioxide	[104]
Aldehyde groups	glyoxyl–agarose	[105]
Polyethyleneimine and glutaraldehyde	Magnetite (PAM) and (TiO_2_)-coated magnetite (TAM)	[106]
Dextran dialdehyde and β-glucosidase-dextran conjugates	silica and aminopropylsilica	[107]
Plasma immersion ion implantation (PIII)	polystyrene films	[108]
	polyethylene granules	[109]

APTMS: 3-Aminopropyl-trimethyoxysilan; CNBr: cyanogen bromide.

**Table 2:** β-glucosidases immobilization by adsorption.

Reactive Group	Immobilization Support	Reference
Physical adsorption	kaolin	[114]
	soil colloidal particles	[115]
	towel gourd vegetable sponges	[116]
Cation Exchanger	Duolite A-568 resin	[117]
	hydroxyapatite (HTP)	[118]
	resin Amberlite DP-1	[119]
	Eudragit S-100	[120]
	polyacrylic resin	[105]
Anion Exchanger	DEAE-sepharose	[121]
	DEAE-cellulose	[122,123]
Anion Exchanger and Macroporous	different ion exchange resins	[124]
Metal Ionic Binding	Magnetic Fe_3_O_4_ nanoparticles coupled with agarose	[125]
	Magnetic Fe_3_O_4_ nanoparticles	[75]
Hydrophobic polyaromatic	Amberlite XAD-4 resin	[126]
	Celite R-640	[127]
Physically immobilized by crossflow ultrafiltration	30 kDa cut-off capillary polysulphone membranes	[128]
	capillary membranes of polysulphone	[129]
Not declared	cellulosic adsorbents: dewaxed, absorbent cotton, CF1 cellulose, Avicel™ PH-101, and Cellufine	[130]

**Table 3:** β-glucosidases immobilized by entrapment methods.

Immobilization Support	Reference
Calcium alginate beads	[70,131,132,133,134,137,139]
Calcium alginate beads and alumina	[95]
Calcium alginate beads and glutaraldehyde	[135,138]
Calcium alginate beads in tetramethoxy-ortho-silicate (TMOS) and hexane	[140]
Calcium alginate and polyacrylamide gel	[136]
Polyacrylamide gel	[102]
Gelatin gel	[141]
Calcium alginate beads, gelatin, polyvinyl alcohol- (PVA-) based matrices (Lentikats), and sol-gel	[142]
Hydrogels of poly(2-hydroxyethyl methacrylate)	[143]
Nanoscale polymeric materials (polyurethane, latex and silicone)	[144]
Ionic liquid sol–gel matrices	[145]
